# Overexpression of *BcERF3* increases the biosynthesis of saikosaponins in *Bupleurum chinense*

**DOI:** 10.1002/2211-5463.13412

**Published:** 2022-05-02

**Authors:** Wenjing Han, Jiao Xu, Hefang Wan, Lei Zhou, Bin Wu, Jianping Gao, Xinwei Guo, Chun Sui, Jianhe Wei

**Affiliations:** ^1^ Institute of Medicinal Plant Development (IMPLAD) Chinese Academy of Medical Sciences & Peking Union Medical College (Key Laboratory of Bioactive Substances and Resources Utilization of Chinese Herbal Medicine, Ministry of Education & National Engineering Laboratory for Breeding of Endangered Medicinal Materials) Beijing China; ^2^ Department of Pharmacognosy Shanxi Medicine University Taiyuan China

**Keywords:** *BcERF3*, biosynthesis of saikosaponin, *Bupleurum chinese* DC., hairy root, overexpression, transcription factor

## Abstract

Chaihu, the dried roots of some species of *Bupleurum* L., is a famous Chinese herbal medicine for treatment of liver‐ and cold‐related diseases, in which saikosaponins (SSs) are the major active compounds. Many of the genetic components upstream of SS biosynthetic pathways have been characterized; however, the regulatory mechanisms remain elusive. In this study we identified the APETALA2/Ethylene Responsive Factor family transcription factor gene *BcERF3* from *B. chinense*. The expression of *BcERF3* was induced in methyl‐jasmonate‐treated adventitious root of *B. chinense*; it was also expressed at higher levels in roots than in other tissues (stem, leaf, flower, and tender fruit of early fruiting plants). Transient expression of *BcERF3* in the leaves of *Nicotiana benthamiana* resulted in intracellular localization of the protein in the nucleus. It was also demonstrated that the number of SSs was greater in *BcERF3‐*overexpressing hairy roots of *B. chinense* than in plants treated with empty vector controls. This coincided with upregulation of *β‐AS*, which encodes a key enzyme involved with triterpenoid biosynthesis. In conclusion, *BcERF3* plays a positive regulatory role in the biosynthesis of SSs.

## Graphical abstract

An AP2/ERF transcription factor, BcERF3, was identified in an important medicinal plant, *Bupleurum chinense*. It is dominantly expressed in the root, where it localizes in cell nuclei and is inducible by MeJA. Overexpression of BcERF3 in *B. chinense* hairy roots caused increased biosynthesis of saikosaponin a, c, and d, accompanied by elevated gene expression of β‐AS (the key enzyme in the biosynthesis of saikosaponin). These findings suggest that BcERF3 plays a positive regulatory role in the biosynthesis of saikosaponin.

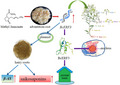

**Abbreviations**
ADS: Amorpha‐4,11‐diene synthase
AP2/ERF: PETALA2/ethylene responsive factor
CBF2: CRTDREHVCBF2
MeJA: methyl‐jasmonate
qRT‐PCR: quantitative real time polymerase chain reaction
RAA: RAV1AAT
SPME: solid‐phase microextraction
SSs: saikosaponins
TF: transcription factor
TIA: terpene indole alkaloid

The AP2/ERF constitutes one of the biggest TF families in plants, which functions play vital roles in plant growth and development [1, 2]. It has been demonstrated that a few of APETALA2/Ethylene Responsive Factor (AP2/ERF) transcription factors (TFs) regulate the biosynthesis of secondary metabolites, including terpenoid, lignan, and alkaloid [3, 4, 5, 6]. Some directly bind to the promoters of structure genes and then regulate the biosynthetic pathway. AaORA, a trichome‐specific AP2/ERF TF from *Artemisia annua*, was identified regulating the expression of *AaERF1*, an enzymatic gene in the artemisinin biosynthetic pathway [7]. AaERF1 and AaERF2 enhanced the biosynthesis of artemisinin by binding to the CRTDREHVCBF2 (CBF2) and RAV1AAT (RAA) motifs present in promoters of both genes of Amorpha‐4,11‐diene synthase (ADS) and CYP71AV1, the two key enzymes of the artemisinin biosynthesis pathway in *A. annua* [8]. CitERF71 activated CitTPS16, a terpene synthase catalyzing the synthesis of E‐geraniol in citrus fruit by directly binding to ACCCGCC and GGCGGG motifs in the promoter [3]. EREB58, a member of the AP2/ERF family in maize, positively regulated sesquiterpene production by directly binding to the GCC‐box within the promoter of TPS10, a terpene synthase [9]. Some AP2/ERF TFs regulated the biosynthesis of secondary metabolites through interaction with other TFs. In *Catharanthus roseus*, two AP2 TFs (ORCA2 and ORCA3) were regulated by methyl‐jasmonate (MeJA)‐inducible CrMYC2 and then regulated the expression of *STR* encoding Strictosidine Synthase in the biosynthetic pathway of terpene indole alkaloid (TIA) [10]. In addition, ORCA4, in an ORCA gene cluster including ORCA2 and ORCA3 in *C. roseus*, showed broad transactivation specificity to a set of TIA genes. ORCA4 overlapped functionally with ORCA3, and unlike ORCA3, ORCA4 overexpression resulted in dramatic increase of TIA accumulation in *C. roseus* hairy roots [4]. In *Isatis indigotica*, AP2/ERF transcription factor Ii049 regulated the synthesis of lignans by binding to the coupling element 1, RAA and CBF2 motifs of the key genes *IiPAL* and *IiCCR* in the biosynthetic pathway [5].

A genome‐wide survey of AP2/ERF TFs revealed more than 100 encoding genes in most plants [11, 12]. According to the conserved AP2 domain (60–70 amino acids residues) and other DNA binding domains, the AP2/ERF family could be divided into five subfamilies, AP2, ERF, DREB, RAV, and Soloist [13]. The members of the ERF and Soloist subfamilies contain one AP2 domain and RAV subfamily members contain an AP2 and a B3 domain. The ERF was the dominant subfamily in plants and were supposed to directly regulate the expression of genes that contained GCC‐box or C‐repeat/DRE cis‐element in their promoter region [14]. AP2 subfamily members contain two AP2 domains. The DREB subfamily contains one AP2 domain. To date, it is not clear whether different subfamilies have their own specific biological functions determined by their difference in domain structure within the AP2/ERF super‐family TFs.

In the genus of *Bupleurum* L., three novel regulatory response factors (ERF), BkERF1, BkERF2.1, and BkERF2.2, were identified from *B. kaoi*. Overexpression of *BkERFs* in transgenic *Arabidopsis thaliana* resulted in elevated mRNA levels of the defense gene *PDF1.2*, and enhanced resistance to *Botrytis* *cinerea* [15]. To date, no AP2/ERF TFs were functionally assayed in *Bupleurum* L. SSs, as the main bioactive component in medicinal species of *Bupleurum* L., is basically accumulated in root cortex with low quantity. The putative biosynthetic pathway of SS was analyzed in detail in our recently published review [16]. The biosynthesis of SSs was highly influenced by the growing environment. It has been noticed that moderate stress could enhance the accumulation of SSs in roots. Investigating the biosynthetic regulation of SSs will be valuable for formulating the agricultural management measures during cultivation to obtain medicinal materials with a high content of SSs. Additionally, hairy root cultivation system have been broadly used to study or produce some bioactive substances. Fully understanding the biosynthesis and regulation of SSs will be a necessary basis for producing SSs by applying hairy root system as well as other metabolic engineering methods. In this study, an AP2 TF was identified from *Bupleurum chinense* functioning in the biosynthesis of SSs, which will drive research on the biosynthetic regulation of SSs.

## Materials and methods

### Gene isolation and sequence analysis of *BcERF3*

Previously, we predicted the TFs by using transcriptome data of *B. chinense* and *B. scorzonerifolium* [17]. The 3′ sequence of *BcERF3* was absent when it was first extracted from the transcriptome data of *B. chinense*. In order to obtain the 3′ sequence of *BcERF3*, the 3′‐RACE PCR (SMARTer RACE cDNA Amplification kit (Clontech, Mountain View, Palo Alto, CA, USA) was conducted to clone its terminal sequences. Finally, the full‐length cDNA sequence was cloned by one round of PCR with reverse‐transcribed root cDNA as ™ template using enzyme of PrimeSTAR HS (Premix) (TaKaRa, Kusatsu, Shiga, Japan). PCR amplification conditions: 94 °C for 5 min; 30 cycles at 94 °C for 30 s, 60 °C for 30 s, and 72 °C for 2 min; a final extension for 10 min at 72 °C; and then cooled to 4 °C. All the primers used in this study are listed in Table S1. The sequence was determined by a sequencing company (Beijing Sunbio Biotech, Beijing, China).

The online ExPASy‐PROSITE web (http://prosite.expasy.org/prosite.html) was used to determine the characteristic domain. The amino acid sequence of BcERF3 was subject to search orthologs using blastp against the nonredundant protein sequences (nr) database (http://www.ncbi.nlm.nih.gov). The hits with high scores were downloaded, and then were used to align and construct a phylogenetic tree using dnaman software (Lynnon Biosoft, San Ramon, CA, USA). The physicochemical parameters of BcERF3 were analyzed using protparam tool (https://web.expasy.org/protparam/).

### *BcERF3* expression in different tissues and MeJA‐treated adventitious roots

Quantitative real‐time polymerase chain reaction (qRT‐PCR) was used to analyze the gene expression. The roots, stems, leaves, flowers, and fruits were dissected from *B. chinense* plants at the early fruiting stage. The adventitious roots of *B. chinense* were cultured using the method in previous reports [17, 18], and MeJA treatment was referenced with the reported method [19]. Briefly, MeJA was added to the freshly prepared liquid media before adventitious roots were transferred. A 200 µm final concentration of MeJA was used for treatment and the adventitious roots were collected at 0 h, 2 h, 4 h, 8 h, 12 h, 24 h, 48 h, and 72 h and 5 days after treatment with non‐MeJA adventitious roots at the same collecting timepoints as controls. All tissues and adventitious roots samples were immediately frozen in liquid nitrogen after collection and then stored at −80 °C for RNA extraction. The protocol of qRT‐PCR was according to a previous report [17]. *Actin* (FJ389747.1) was used as the internal reference gene of qRT‐PCR for the MeJA treatment experiment, and *β‐tubulin* (FJ389750) for the different organ expression experiment. For all qRT‐PCR assaya, three replicates were used. All column bar figures were made using graphpad prism 6.0 software (GraphPad Software, San Diego, CA, USA).

### Subcellular localization of BcERF3

Both recombinants, pGD‐EGFP‐BcERF3 and pGDSRED2‐BcERF3, in which the BcAP2 encoding sequence was ligated in fusion with green and red fluorescent proteins, respectively, were constructed and transformed to *Escherichia coli* strains DH5α and then were transformed into *Agrobacterium tumefaciens* GV3101 after sequencing verification. The empty vector GD‐EGFP‐ and pGDSRED2‐transformed GV3101 cells were used as control. *Nicotiana* *benthamiana* plants were used for agroinfiltration experiments according to a previous report [20]. After agroinfiltration, plants were maintained in the incubator under continuous fluorescent lighting for 24 h and then were transferred to a growth chamber at 22 °C with a 16 h/8 h light/dark photoperiod. Leaves were examined by confocal microscopy (Bio‐Rad, Hercules, CA, USA) after 72 h postinfiltration.

### *BcERF3* overexpression in hairy roots

The recombinant plasmid pK2GW7‐BcERF3 was conducted in which the BcAP2 encoding sequence was inserted into the expression vector pK2GW7. Both pK2GW7‐BcERF3 and pK2GW7 were separately transformed to *Agrobacterium rhizogenes* SW101, and then were subject to infecting the tender leaves of sterile seedlings of *B. chinense*. The hairy roots started to develop after about 2 weeks of cultivation. Hairy roots about 2 cm were excised from explants and cultured in fresh MS culture medium containing 500 mg·L^−1^ cefotaxime in the dark. After about 4 weeks, hairy roots were transferred to MS liquid medium cultured in the dark with shaking (150 r.p.m.), until the total quantity of each independent line was enough for chemical components analysis. The hairy root lines induced by *A. rhizogenes* carrying empty pK2GW7 were used as control. The overexpression of *BcERF3* was verified by qRT‐PCR; meanwhile, the expression of *β‐AS,* which encodes β‐amyrin synthase, was tested. *Actin* was used as the internal reference gene and the $2^{‐\Delta \Delta C_{t}}$ method was used to calculate the relative expression of genes. Three replicates were assayed.

### HPLC detection of SSs in BcERF3‐overexpressing hairy root lines

Four independent lines overexpressing *BcERF3* were used to analyze the content of SSs (the total content of three major SS monomers, SS a, SS c, SS d). Commercial SS a, c, d were used as reference standards. Fresh hairy roots were freeze‐dried in a freeze‐dryer, ground into a fine powder with a mortar, and passed through a 60‐mesh sieve. Accurately weighed 0.5 g of the powder was put into a 100 mL conical flask and 25 mL of 5% ammonia methanol solution was added for ultrasonic extraction for 35 min. Two extractions were conducted. Filtered with a funnel, all the extracts were placed in a 70 °C water bath to evaporate to dryness and then were dissolved to 5 mL with 20% methanol solution. The solid‐phase microextraction (SPME) column Cleanert S C18 (Bonna‐Agela, Tianjin, China) was used for separation. The eluted solution was nitrogen blow‐dried and dissolved into 500 μL methanol for assay with HPLC using Waters 2487 (Waters, Shanghai, China). Column (ASB‐vensil C18 column: 25 cm × 4.6 mm, 5 µm) was used; The mobile phase A was 5‐mm ammonium acetate solution, and mobile phase B was acetonitrile (chromatographic grade); Flow rate was 1.0 mL·min^−1^; Injection volume was 50 μL; Column temperature was 30 ± 0.5 °C. The elution program was: 0–18 min, 10–85% B; 18–22 min, 85% B; 22–32 min, 85–70% B; 32–42 min, 70–50% B; 42–52 min, 50–10% B; and 52–60 min, 10% B. The quantification of SS a, c, d was calculated by comparison with reference standards. Three replicates for each line were assayed.

## Results

### Gene isolation and expression analysis of *BcERF3*

**Fig. 1:**
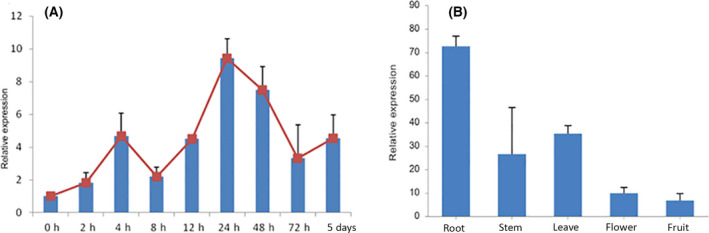
Expression patterns of *BcERF3* after exposure to MeJA and in different tissues of *Bupleurum chinense*. (A) Expression profile of *BcERF3* in adventitious roots that were collected at different times post‐MeJA (200 µm) treatment. (B) Expression pattern of *BcERF3* in different tissues, root, stem, leave, flower, and fruit. Bar graphs represent mean with SD, *n* = 3.

The full‐length cDNA sequence of *BcERF3* was obtained with the aid of 3′‐RACE PCR. The open reading frame of BcERF3 was 600 bp encoding 199 amino acids (Table 1). The expression of *BcERF3* was assayed in MeJA‐treated adventitious roots and organs of *B. chinense* by real‐time qRT‐PCR. It was shown that the expression of *BcERF3* was induced by MeJA and the transcript reached the highest level at 24 h after MeJA was added to the media. After 24 h, the induction effect of MeJA on the expression of *BcERF3* decreased; however, the expression levels were still significantly higher in MeJA‐treated adventitious roots than in non‐MeJA adventitious roots. Meanwhile, it could be seen that MeJA‐treated adventitious roots showed a slower growth rate than non‐MeJA adventitious roots (Fig. 1A). In addition, *BcERF3* was dominantly expressed in roots of early fruiting plants of *B. chinense* (Fig. 1B).

**Table 1:** Physicochemical parameters of *BcERF3*.

Parameters		*BcERF3*	
Molecular weight		22449.07 Dalton	
No. of amino acids		199	
Theoretical pI		5.84	
Instability index (II)		68.93	
No. of negatively charged residues (Asp + Glu)		31	
No. of positively charged residues (Arg + Lys)		29	
Aliphatic index		70.15	
Grand average of hydropathicity (GRAVY)		−0.633	
Formula		C_972_H_1540_N_286_O_312_S_7_	
**Amino acid composition**			
Ala (A)	20 (10.1%)	Lys (K)	10 (5.0%)
Arg (R)	19 (9.5%)	Met (M)	5 (2.5%)
Asn (N)	9 (4.5%)	Phe (F)	7 (3.5%)
Asp (D)	14 (7.0%)	Pro (P)	8 (4.0%)
Cys (C)	2 (1.0%)	Ser (S)	23 (11.6%)
Gln (Q)	4 (2.0%)	Thr (T)	8 (4.0%)
Glu (E)	17 (8.5%)	Trp (W)	3 (1.5%)
Gly (G)	8 (4.0%)	Tyr (Y)	6 (3.0%)
His (H)	2 (1.0%)	Val (V)	13 (6.5%)
Ile (I)	7 (3.5%)	Pyl (O)	0 (0.0%)
Leu (L)	14 (7.0%)	Sec (U)	0 (0.0%)

### Bioinformatics analysis of BcERF3

**Fig. 2:**
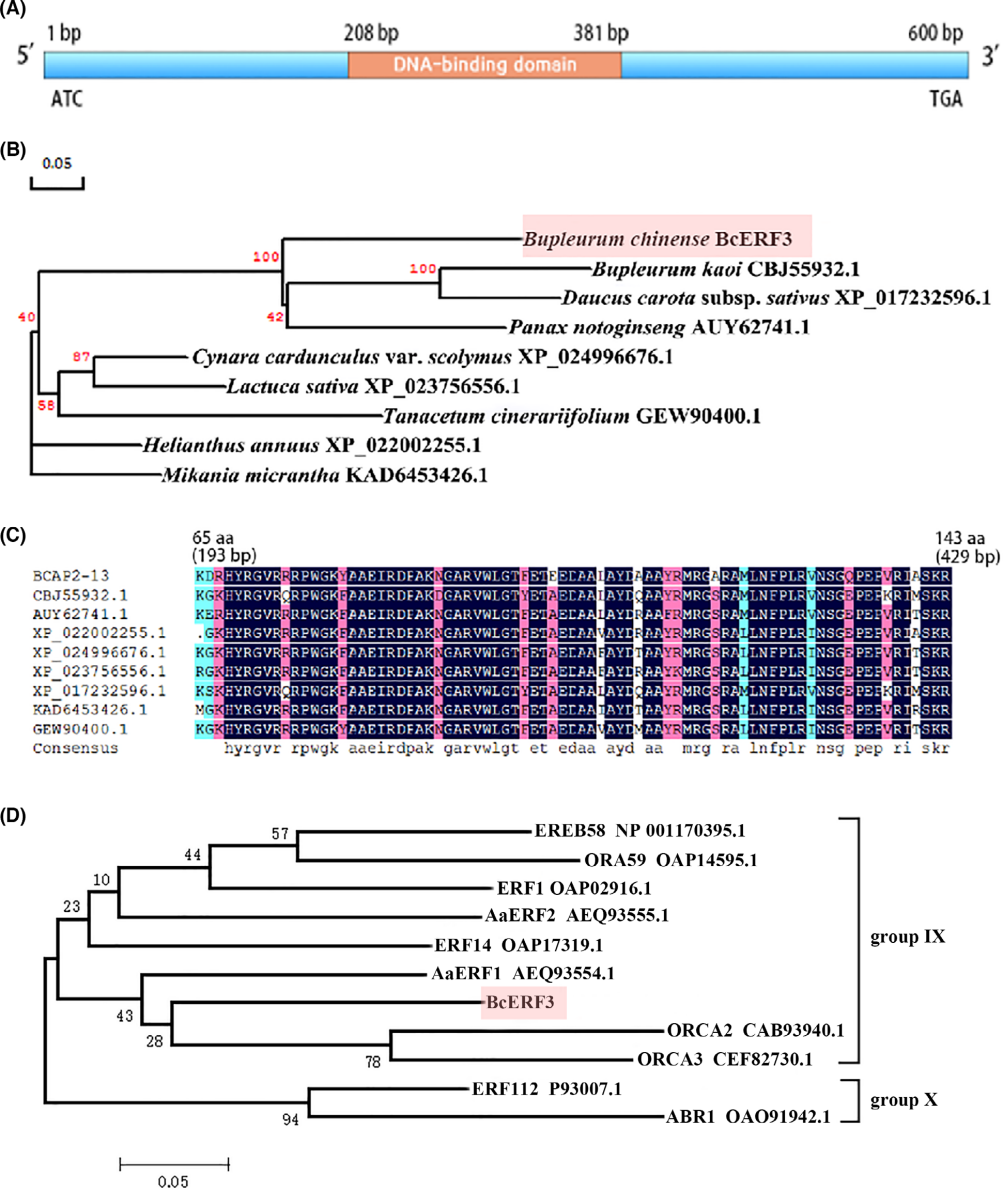
Sequence analysis of BcERF3. (A) The open reading frame structure of *BcERF3*. Pink part represents the conserved DNA binding domain of the AP2 superfamily. (B) The phylogenetic tree of BcERF3 and its homologous proteins from other plants constructed using dnaman software with Maximum Likelihood distance and 1000 bootstrap repeats. (C) Comparison of conserved amino acids of BcERF3 and other homologous proteins. The source plant species of the GenBank numbers in C are shown in B. (D) Comparison of the amino acid sequence of BcERF3 with other functionally known AP2/ERF TFs using MeGA software.

Conserved domain analysis showed that the BcERF3 protein contained one characteristic domain of AP2, implying that it might be a member of AP2/ERF TFs (Fig. 2A). The homologous proteins of BcERF3 were searched in NCBI nonredundant protein sequences database by the web blastx tool. The result showed that BcERF3 shared the highest sequence identity (59%) with an ethylene‐responsive transcription factor 2‐like protein from *Daucus carota* subsp. *sativus* (XP_017232596.1), followed by an AP2/ERF TF from *Panax notoginseng* (AUY62741) with 56% identity, and the ethylene response factor 2.1 from *B. kaoi* (CBJ55932.1) with 53% identity (Fig. 2B). Furthermore, a partial sequence of BcERF3, which was about from 40 aa to 140 aa, shared more than 70% identity with several ethylene‐responsive transcription factor‐like proteins from diverse plant species (Fig. 2C). In addition, the amino acid sequence of BcERF3 was compared with other functionally known AP2/ERF TFs, showing that BcERF3 was most closely related to ORCA2 and ORCA3, which belong to the group IX of ERF (Fig. 2D).

### Subcellular localization of BcERF3

**Fig. 3:**
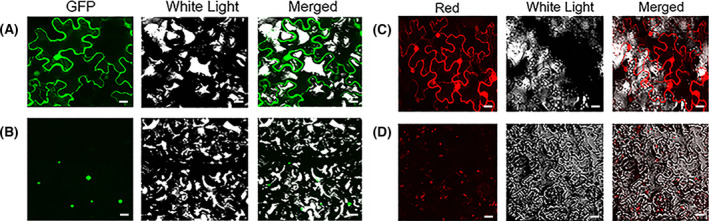
Subcellular localization of BcERF3 protein. (A,C) The *Nicotiana* *benthamiana* leaf cells were agroinfiltrated with pGD‐EGFP and pGDSRED2 empty vectors, respectively. The green or red fluorescence emitted in the whole cells. (B,D) The *N. benthamiana* leaf cells were agroinfiltrated with pGD‐EGFP‐BcERF3 and pGDSRED2‐BcERF3 recombinant vector, respectively. The green or red fluorescence exclusively emitted in the nuclei of cells. Scales bars = 20 µm in (A–D).

The targeting experiment was conducted using fusion vectors with green or red fluorescence emission signatures and the well‐established tobacco leaves agroinfiltration system. As illustrated in Fig. 3, the BcERF3‐GFP fusion protein was mainly detected in the nuclei, while the GFP protein alone exhibited a uniform distribution throughout the tobacco cells (Fig. 3A,B). Similarly, the red fluorescence could be exclusively detected in the nuclei of pGDSRED2‐BcERF3 agroinfiltrated cells, while the red fluorescence spread through the entire pGDSRED2 agroinfiltrated cells (Fig. 3C,D). Therefore, it was confirmed that BcERF3 was located in nuclei where it might function as a TF regulating the expression of its target genes.

### Overexpression of BcERF3 in the hairy root of *B. chinense*

**Fig. 4:**
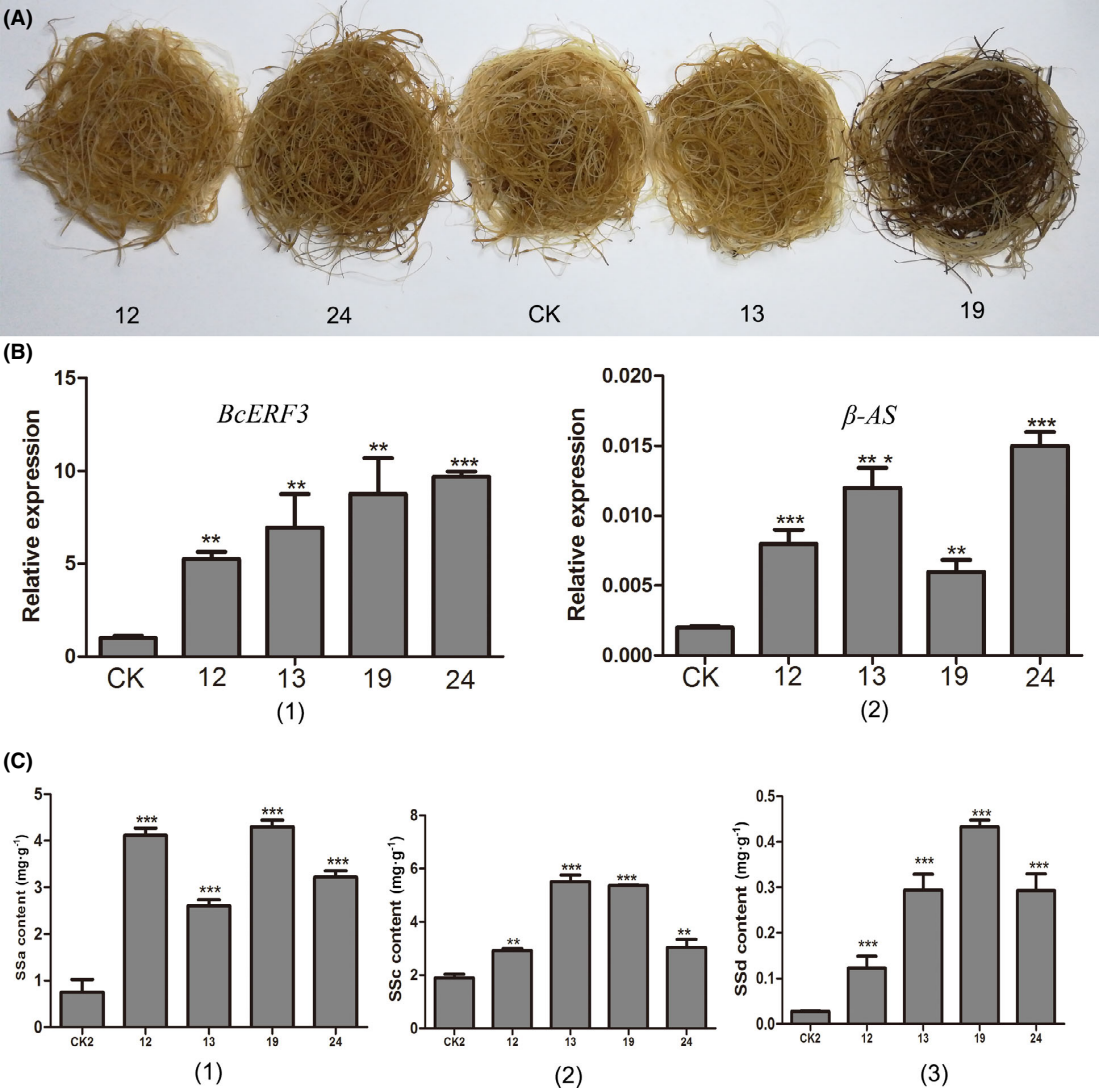
Analysis on the transgenic lines overexpressing *BcERF3*. (A) *BcERF3* and pK2GW7 empty vector transformed hairy roots of *Bupleurum chinense*. (B) The expression of *BcERF3* and *β‐AS* in transgenic lines assayed by real‐time qRT‐PCR showing higher expression levels in *BcERF3* transformed lines. (C) The content of SS a, c, and d in transgenic lines assayed by HPLC showing elevated biosynthesis of SSs in *BcERF3* transformed lines. Bar graphs represent mean ± SD, *n* = 3. Student's *t* test, *P < 0.05, **P < 0.01, ***P < 0.001.

Transgenic hairy roots of *B. chinense* overexpressing *BcERF3* were obtained. The overexpression of *BcERF3* was confirmed by real‐time qRT‐PCR. Four positive lines were further analyzed (Fig. 4A). Observing the hairy root appearance of the four lines, Line 19 obviously showed the darkest color when compared with CK and three other lines, and Line 24 showed a little bit darker than CK, Line 12 and 13. There was no obvious difference between CK, Line 12 and Line 13. The expression of *BcERF3* showed the highest level in Line 24 with about 8‐fold of CK and the lowest level in Line 12 with about 4‐fold of CK (Fig. 4B). Meanwhile, the expression levels of *β‐AS* in the four overexpression lines and CK were measured. Compared with CK, all the four overexpression lines showed higher expression levels of *β‐AS* with more than 3‐fold (Fig. 4B). The contents of SS a, c, and d were determined by HPLC. The results showed that the biosynthesis of three SS monomers were all elevated in the four overexpression lines. The highest content of SS a, c, and d in total was found in Line 19 (Fig. 4C).

## Discussion

Sequence analysis showed only one specific DNA‐binding domain of AP2/ERF existed in BcERF3. In addition, ERF subfamily characteristic amino acids, alanine in position 14 and aspartic acid in position 19, could be found in a specific domain [21]. It could be deduced that BcERF3 belonged to the ERF subfamily of AP2/ERF TFs. In general, AP2 subfamily members with two specific domains played vital roles in different plant organs development, while the ERF subfamily members mainly participated in a plant stress response. The gene expression of ERF subfamily TFs usually could be affected by biotic and abiotic stresses or phytohormones induction, indicating that these genes functioned in different signal transduction pathways. As crosstalk factors, the vital roles of ERF subfamily TFs in stress response and signal transduction have been previously characterized, but some questions remain [22]. We found that the expression of *BcERF3* was induced by MeJA, an important regulator in plant response to biotic and abiotic stresses. In fact, several ERF TFs that regulated the biosynthesis of terpenoids were responsive to MeJA treatment [23, 24]. More work will be needed to resolve the precise metabolic network underlying phytohormones signal transduction and terpenoids biosynthesis.

Combining transcriptome or genome sequencing and overexpressing target genes in transgenic hairy roots have shown to be an efficient strategy for functional verification of interesting genes. It has been used in most studies on the biosynthetic pathway and regulation of metabolites, such as tanshinone and phenolic acids in *Salvia miltiorrhiza* [6, 24, 25], terpenoid indole alkaloids in *C. roseus* [23] and ginsenoside in *P. ginseng* [26]. In this study, *BcERF3* was overexpressed in *B. chinense* hairy roots and four lines were analyzed. Of note, Line 19 showed a darker color in appearance than the other lines. The contents of SS a, c, and d in Line 19 were all relatively higher than in other overexpressed lines. But the expression of *β‐AS* encoding the key biosynthetic enzyme of SS was not the highest among all overexpressed lines, although it was significantly higher than the control. The gene expression level of *BcERF3* and *β‐AS* in Line 24 was the highest among all tested lines. We found Line 19 grew faster than others and it first reached a saturated quantity for the volume of the culture flask. It was possible that the limited space and oxygen led to the dark color roots and restrained fresh rootlets. Most root segments of Line 19 grew for more time than the others when all lines were collected at the same time. So, it could be deduced that more SS a, c, and d accumulated in older root segments and more active biosynthetic reaction happened in younger rootlets, which may explain why the highest SSs content was obtained in Line 19 rather than Line 24.

Based on its dominant expression in roots and inducible expression by MeJA, *BcERF3* was firstly predicted involving with the biosynthesis of SSs in *B. chinense*. Overexpression of *BcERF3* in hairy roots of *B. chinense* further confirmed its regulatory function on the biosynthesis of SSs, since a significant content elevation was found in transgenic lines. SS a, c, and d were the main SS monomers, with clearly higher contents than others in *B. chinense* and were found in all existing species of the *Bupleurum* genus. The three kinds of SS monomers shared the same aglycone of type I that was 13β and 28‐epoxyolean‐11‐ene‐16‐ol [27]. The putative biosynthetic pathway of SS was reviewed, and it underwent through the isoprenoid pathway, the cyclization of oxidosqualene, and some modifications such as oxidation, glycosylation [28]. In our study, the biosynthesis of SS a, c, and d were all enhanced in the four *BcERF3* overexpressed hairy root lines, meaning the regulatory target may be any of the genes of enzymes that participated in the biosynthesis of type I aglycone of SSs. It will accelerate the understanding on biosynthesis of SSs in *Bupleurum* when the exact regulatory target of BcERF3 is revealed.

## Conflict of interest

The authors declare no conflicts of interest.

## Author contributions

WH prepared the article. JX conducted experiments. HW and LZ were involved in article preparation. BW, JG, and XG provided suggestions for the experiments. CS designed the experiments and checked the article. JW provided suggestions for designing the experiments. All authors reviewed and approved the final version and agreed to be held accountable for the article content.

## Supporting information


**Table S1.** All primers used in this study.

## Data Availability

Raw data are available from the corresponding author upon reasonable request.
