# Supplementary material for: Overexpression of BcERF3 increases the biosynthesis of saikosaponins in Bupleurum chinense
Source: FEBS Open Bio. 2022 May 2;12(7):1344–52. doi: 10.1002/2211-5463.13412 (PMC9249337; doi:10.1002/2211-5463.13412)
Supplement: Supplementary file 1 — Table S1. All primers used in this study. [file FEB4-12-1344-s001.doc]

**Supporting information**

| **Table S1.** All primers used in this study |  |  |  |
| --- | --- | --- | --- |
| Purposes | Genes | Primers | Sequences (5'-3') |
| 3' RACE | *BcERF3* | *BcERF3-GSP3* | TGTCGTCGGATTCGTCAGAGGCGGTGGT |
|  |  | *BcERF3-NGSP3* | TCAGAGGCGGTGGTGGCGAAGCG |
| Full-length cDNA cloning | *BcERF3* | *BcERF3-F* | CATCATTCCACTACAAAGACAC |
|  |  | *BcERF3-R* | AATAAAATAAGTCCAAGGCGTGAAA |
| Subcellular localization | *BcERF3* | *BglII-13-F* | GGACGTAGATCTATGATGCAATCAAATTTTGG |
|  |  | *HindIII-13-R* | CCCAAGCTTGAACAAGACAATTAAAAAAGTC |
| Overexpression vector construction | *BcERF3* | *G-13-F* | GGGGACAAGTTTGTACAAAAAAGCAGGCTTA  ATGATGCAATCAAATTTTGGA |
|  |  | *G-13-R* | GGGGACCACTTTGTACAAGAAAGCTGGGTAT  CAAACAAGACAATTAAAAAAGTCG |
| Verification of transformed hairy root lines | *nptII* | *P-35SP* | TCTACCCGAGTAATAATCTCCAGG |
|  |  | *P-35ST* | CGAAGGATAGTGGGATTGTGC |
| Expression analysis | *BcERF3* | *BcERF3-F* | GTCAAGGACAGGCATTACAGA |
|  |  | *BcERF3-R* | AAGCAGCAGCATCATACGC |
|  | *Actin* | *BkActinPf* | TCACCATTGGAGCTGAGAGATTC |
|  |  | *BkActinPr* | CTGCAGCTTCCATTCCAATCA |
|  | *β-tubulin* | *Tubulin-F* | ATGTTCAGGCGCAAGGCTT |
|  |  | *Tubulin-R* | TCTGCAACCGGGTCATTCAT |
|  | *β-AS* | *β-AS-F* | F-ACATGGCTTTCGATACTCGG |
|  |  | *β-AS-R* | R-ATTTTCGCTGGATGCATAGG |
